# Could motor unit control strategies be partially preserved after stroke?

**DOI:** 10.3389/fnhum.2014.00864

**Published:** 2014-10-31

**Authors:** S. Jayne Garland, Courtney L. Pollock, Tanya D. Ivanova

**Affiliations:** ^1^Department of Physical Therapy, University of British ColumbiaVancouver, BC, Canada; ^2^Graduate Program in Rehabilitation Sciences, Department of Physical Therapy, University of British ColumbiaVancouver, BC, Canada

**Keywords:** motoneuron, stroke, common drive, afterhyperpolarization, posture

## Introduction

There is considerable evidence on the impairments that a cerebral stroke will have down-stream of the stroke, i.e., in the spinal motoneuron and the muscle. Motor impairment following stroke has been documented as force production that is slow, weak, and lacking in precision (Garland et al., 2009) and is associated with difficulty in fully activating the muscle (Klein et al., 2013). Furthermore, in functional tasks such as standing balance and gait, there is evidence of deficits in intra-limb coordination of muscles even on the non-paretic side (Marigold and Eng, 2006; Raja et al., 2012). In this opinion paper, we will first briefly review the changes observed at the level of the motor unit (MU) after stroke and second reflect upon whether some changes in the intrinsic properties of motoneurons, typically considered to be maladaptive, might also reflect a positive adaptation that could assist in force production. Lastly, this paper will explore the control of MUs between limbs during standing balance and suggest that, while some impairment may exist, there remains the possibility of a preservation of fundamental motor control strategies after stroke that might be a target for rehabilitation.

## Motor unit/muscle characteristics

At the level of the MU, studies have demonstrated a loss of spinal motoneurons following stroke (McComas et al., 1973; Hara et al., 2004; Lukacs, 2005; Li et al., 2011), particularly those that innervate type II MUs (Lukacs et al., 2008). It has been suggested that chronically paretic muscle is made up of fewer, but larger, MUs due to collateral sprouting of the remaining motoneurons to innervate a greater number of muscle fibers (Lukacs, 2005; Kallenberg and Hermens, 2011; Li et al., 2011) and this process could result in a mismatch of muscle fiber type and motoneuron characteristics (Young and Mayer, 1982; Dattola et al., 1993). Ultimately both of these changes may result in muscle contractions with slower rates of force development and decreased levels of force production (Garland et al., 2009).

In addition to the MU remodeling described above, MU discharge characteristics have been observed to change following stroke. For instance, MU firing rates in the paretic muscle are lower than the non-paretic muscle during ramp isometric contractions (Gemperline et al., 1995; Frontera et al., 1997; Chou et al., 2013; Mottram et al., 2014). It has also been demonstrated that the range of modulation of firing rates is compressed in paretic muscle, such that the increase in firing rate in response to increased force production is limited (Gemperline et al., 1995; Frontera et al., 1997) and the modest increases in firing rate saturate with higher forces (Mottram et al., 2014). Chou et al. (2013) observed that the decreased MU firing rate was associated with a decreased rate and magnitude of force production, suggesting that the limitation in firing rate offered a potential mechanism for the decreased speed of voluntary movement in people post-stroke.

Most of the MU research has been performed in chronic stroke survivors and hence changes would be expected at the level of the muscle when disuse could be a contributing factor. Gray et al. (2012) summarized that chronic stroke results in a decrease in muscle mass, decrease in fiber length, and a smaller pennation angle which would affect the paretic muscle's ability to generate force. A systematic review and meta-analysis performed by English et al. (2010) demonstrated that lean paretic muscle mass was significantly less than that of non-paretic muscle, in both upper and lower extremities, in people at least 6 months post-stroke. However, Klein et al. (2013) and Ramsay et al. (2011) showed that the tibialis anterior (TA) muscle was not atrophied in people after chronic stroke, despite the prevalence of foot-drop in gait (Kesar et al., 2010). Ramsay et al. (2011) also demonstrated that in the plantarflexor complex, the gastrocnemii muscles show significantly greater atrophy than the soleus muscle. Considering the differences in function and muscle morphology (TA and soleus have fewer fast twitch MU than gastrocnemii; Johnson et al., 1973) across the muscles, the impact of MU loss and remodeling following stroke may be muscle- or task-dependent and could explain differences across studies.

## Motoneuron intrinsic properties

One way to examine the intrinsic properties of the motoneuron in humans is to use the Interval Death Rate (IDR) analysis, which estimates the time-course of the motoneuron afterhyperpolarization (AHP) based on the discharge pattern of the MUs (Matthews, 1996). The duration of the AHP is important because it influences the discharge rate of the MU (Bakels and Kernell, 1993a,b; MacDonell et al., 2008). MU size and parameters of the AHP have been shown to be inversely related to each other in mammalian models; that is, the greater the amplitude of the AHP and the longer the AHP half decay time, the smaller the MU (Powers and Binder, 2001). The IDR model has been used in humans to show the relationship of the contractile properties of an intrinsic hand muscle to the AHP time-course (Gossen et al., 2003). This “speed-match” between the time course of the motoneuron AHP and the time course of its muscle unit twitch is thought to be functionally-relevant so that the minimal firing rate of the MU is matched to the twitch contraction properties (Gardiner and Kernell, 1990).

We performed the IDR analysis in TA of young healthy subjects and also in people after stroke. While the AHP time constant estimated on the non-paretic side (36.2 ± 6.4 ms, Ivanova et al., 2014) was consistent with that of young healthy individuals (32.9 ± 4.4 ms, MacDonell et al., 2008 and 33.6 ± 4.5 ms, Christie and Kamen, 2010), and older participants (37.3 ± 4.7 ms, Christie and Kamen, 2010), the AHP decay time-constant was significantly prolonged on the paretic side (41.7 ± 8.5 ms, Ivanova et al., 2014). Significant prolongation of the AHP time course after stroke in the upper extremity motoneurons was also reported by Liang et al. (2010) for the biceps brachii in participants with spasticity awaiting botox injection and by Suresh et al. (2014) for the first dorsal interosseous muscle. Our work revealed that the AHP time constants in TA were significantly longer in the low recovery group than in the high recovery group, indicating a relationship between the severity of motor impairment and AHP prolongation (Ivanova et al., 2014), albeit such a relationship was not found in a small hand muscle (Suresh et al., 2014). Nevertheless, Suresh et al. (2014) did suggest that the longer duration of the AHP contributed to the lower MU discharge rate in the paretic muscle after stroke. In the rat, prolongation in the AHP has been observed with chronic spinalization (Bennett et al., 2001) or tetrodotoxin-induced paralysis (Cormery et al., 2000), both being examples of motoneuron plasticity accompanying muscle disuse.

Given the above findings, it is clear that the motoneuron AHP is prolonged in the presence of chronic stroke and this may contribute to the limitations in MU firing rate and motor impairments observed after stroke. It is important to note however, that although there is *on average* a prolongation of AHP after stroke, there is also substantial overlap in AHP time-constants with that found in healthy subjects. What is not clear is whether the AHP prolongation might reflect positive adaptations of the motoneuron to maintain the “speed match.” Deficits in velocity of movement are well-documented after stroke (Bohannon, 1987; Davies et al., 1996; Lum et al., 2004). Could the motoneuron be adapting to the remodeling at the level of the muscle? No studies have yet been performed to determine if the time-course of the AHP and the muscle unit remain matched after stroke. These experiments would need to be performed to determine if the adaptations of the motoneuron and firing rate represent a necessary compensation to the muscle changes.

## Common modulation of motor unit during standing

While the previous section discussed the way in which MUs might control force within a muscle, the motor control of functional tasks like standing require the coordination of MU activity between the limbs. Earlier work from our laboratory explored the role of soleus muscles bilaterally in the postural control of standing by quantifying MU synchronization, common drive and coherence (Mochizuki et al., 2005, 2007). Although soleus MUs within a single muscle had modest levels of synchronization during quiet stance, the incidence of synchronization between the soleus muscles bilaterally was very low (Mochizuki et al., 2005). In contrast, Gibbs et al. (1995) showed evidence of synchronization in 7/10 healthy subjects between bilateral gastrocnemius muscles during a demanding balancing task, possibly revealing a task- or muscle-dependent response. The strength of common modulation of MU discharge was greater in unilateral than bilateral MU pairs, and greater during postural tasks than voluntary isometric tasks (Mochizuki et al., 2007). Altering proprioceptive input by vibrating one leg resulted in a significant reduction in common drive, suggesting that sensory input contributes to the common modulation of MU discharge during postural tasks (Mochizuki et al., 2007). Given the sensorimotor impairments after stroke, one would expect a significant disruption in the common modulation between the paretic and the non-paretic limb.

**Figure 1:**
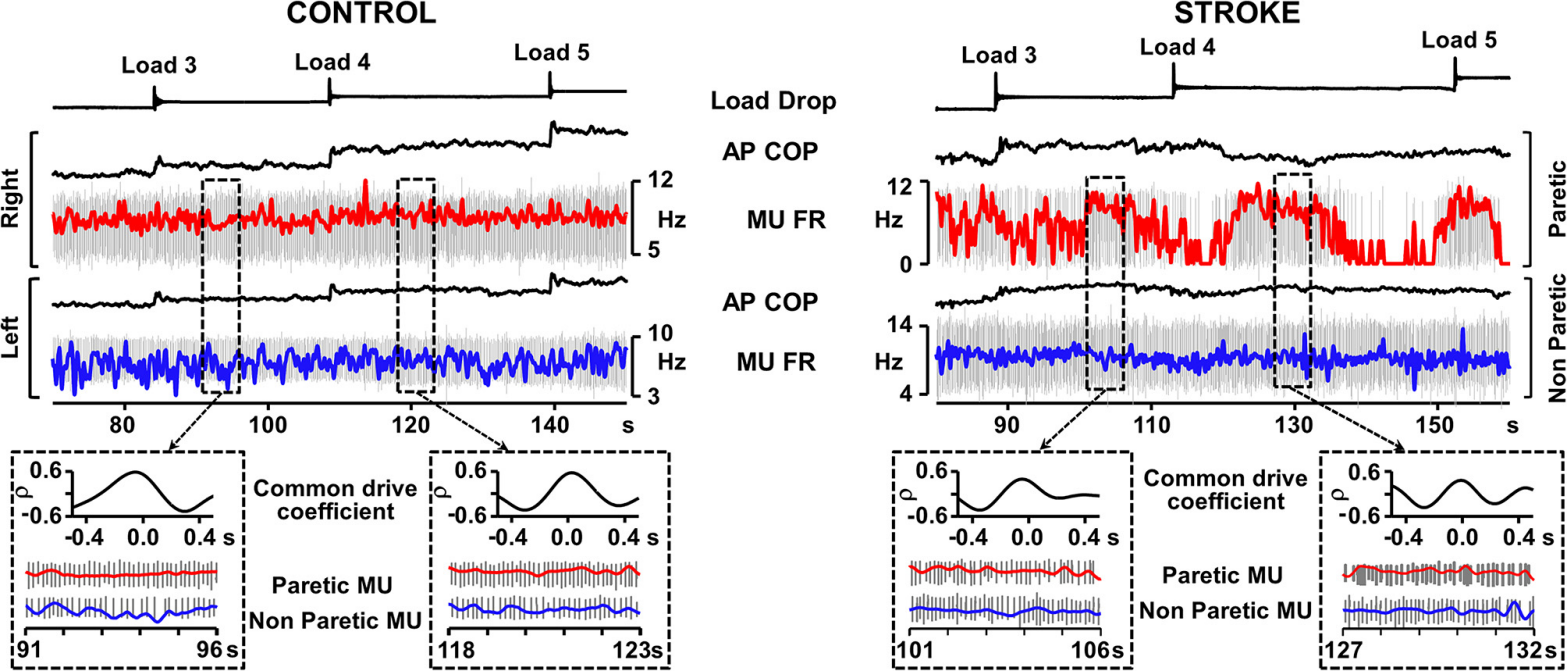
**Representative recordings from Control and Stroke participants demonstrating common modulation of firing rate**. Traces from top; load drop, anterior-posterior center of pressure (AP COP), and smoothed motor unit (MU) firing rate superimposed on motor unit action potentials (red trace, right leg control, paretic leg), AP COP and MU firing rate (FR) superimposed on MU trains (blue trace, left leg controls, non-paretic leg). Dashed boxes outline examples of 5 s epochs where both motor units in the pair were firing steadily and the common drive coefficient was calculated. Cross-correlograms and expanded motor unit firing rate and action potential traces are presented for each of the outlined epochs. Despite the difficulty in maintaining consistent MU firing rate in the paretic leg, the epochs in which the MU was firing steadily rendered common drive coefficients that were similar to control.

Whereas the soleus muscle demonstrates tonic EMG activation in standing, the EMG activity of the medial gastrocnemius muscle is more responsive to anterior displacement of the center of pressure (COP) (Di Giulio et al., 2009). We examined the common drive in people after stroke in the medial gastrocnemius muscle during an external perturbation task in standing. Eleven experiments in six participants in the chronic stage after stroke (time post-stroke, 6.8 ± 3.8 years) with mild to moderate levels of motor impairment (Chedoke-McMaster Stroke Assessment foot score, 4.3 ± 1.5/7) and 5 experiments in 5 healthy controls were conducted. A belt placed around the hips was attached to a horizontal cable in front of the subject. Loads were applied by dropping weights of 1% body mass into a basket on the end of the cable every 30 s until a maximum of 5% body mass was in the basket. This resulted in a gradual progression of the anterior COP (Figure 1). During the 25–30 s between load drops, MUs that were firing steadily were analyzed. Common drive analysis involved converting MU spike trains to continuous firing rate signals (De Luca et al., 1982). The signals were then high-pass filtered and cross-correlated over a moving window of 3–5 s epochs (Monsour et al., 2012) to produce a common drive coefficient (rho) that was averaged across epochs.

Two-Way ANOVA analysis (with Tukey *post-hocs*) revealed that common drive in MU pairs derived from bilateral muscles (Figure 1) was significantly lower in people after stroke (15 MU pairs, rho = 0.44 ± 0.13; mean ± SD) than controls (17 MU pairs, rho = 0.58 ± 0.06, *p* < 0.01), as well as for unilateral MU pairs in the paretic muscles after stroke (10 MU pairs, rho = 0.67 ± 0.07) than controls (12 MU pairs, rho = 0.77 ± 0.06, *p* < 0.01). However, the level of common drive *within* the paretic medial gastrocnemius muscle remained relatively high and there was a moderate level of common drive *bilaterally* after stroke indicating that MUs in the paretic muscle still co-modulated with the non-paretic side. Given the range of common drive coefficients, there were MU pairs after stroke that exhibited the same amount of common modulation as that found in healthy persons (Figure 1). These findings suggest that MU control strategies such as common drive during postural tasks, while diminished, remain present after stroke.

## Concluding remarks

There is no doubt that there are changes in the MU discharge characteristics after stroke. But the AHP and common drive data suggest that residual motor control strategies may remain after stroke, albeit diminished, and may reveal a need to consider functional task-dependency in future research to explore MU impairment and adaptation post-stroke. It remains to be seen whether treatments that challenge the neuromuscular system could prevent the muscle remodeling and any compensatory MU control adaptations.

### Conflict of interest statement

The authors declare that the research was conducted in the absence of any commercial or financial relationships that could be construed as a potential conflict of interest.
